# Membrane Nanowaves in Single and Collective Cell Migration

**DOI:** 10.1371/journal.pone.0097855

**Published:** 2014-05-20

**Authors:** Omar F. Zouani, Veronika Gocheva, Marie-Christine Durrieu

**Affiliations:** 1 Bioingénierie Tissulaire (BioTis), INSERM U1026, Université de Bordeaux, Bordeaux, France; 2 Institut Européen de Chimie et Biologie (IECB), CNRS, UMR 5248, Université de Bordeaux I, Pessac, France; 3 Université Montpellier II, Montpellier, France; The University of Akron, United States of America

## Abstract

We report the characterization of three-dimensional membrane waves for migrating single and collective cells and describe their propagation using wide-field optical profiling technique with nanometer resolution. We reveal the existence of small and large membrane waves the amplitudes of which are in the range of ∼3–7 nm to ∼16–25 nm respectively, through the cell. For migrating single-cells, the amplitude of these waves is about 30 nm near the cell edge. Two or more different directions of propagation of the membrane nanowaves inside the same cell can be observed. After increasing the migration velocity by BMP-2 treatment, only one wave direction of propagation exists with an increase in the average amplitude (more than 80 nm near the cell edge). Furthermore for collective-cell migration, these membrane nanowaves are attenuated on the leader cells and poor transmission of these nanowaves to follower cells was observed. After BMP-2 treatment, the membrane nanowaves are transmitted from the leader cell to several rows of follower cells. Surprisingly, the vast majority of the observed membrane nanowaves is shared between the adjacent cells. These results give a new view on how single and collective-cells modulate their motility. This work has significant implications for the therapeutic use of BMPs for the regeneration of skin tissue.

## Introduction

Cell migration within a tissue is a fundamental biological process. It is essential for organ regeneration [1] and wound healing but is also involved in certain diseases like cancer metastasis [2]–[4]. The mechanism of cell migration involves membrane ruffling at the leading cell edge that is rapidly induced in response to certain extracellular signals. Membrane ruffling is characterized by dynamically fluctuating movements of membrane protrusions like blebs, lamellipodia and filopodia driven by dynamic rearrangements of cytoskeleton components beneath the plasma membrane [5]–[7]. Although many aspects of the molecular mechanisms of cell motility are still not clear accumulating evidence indeed suggests that certain growth factors like the platelet-derived growth factor (PDGF) and the bone morphogenetic proteins (BMPs) [8]–[11] are required. They could activate the Rho GTPases like Rac1 and Cdc42 [12] and thus control the lamellipodia formation and membrane ruffling via regulation of the polymerization and depolymerization of the actin filaments.

Very interestingly, membrane waves were described in the recent years and introduced as a new mechanistic component in the understanding of cell motility [13]–[16]. In fact, cells have the ability to produce centripetally propagating waves on their membranes, which are traveling membrane undulations that persist over microns. These waves are believed to be driven by the interactions of motile proteins like actin and myosin associated with the cell membrane. Such membrane waves have been observed in a variety of cells [13], [17], [18]. For example, on fibroblasts, the amplitudes of these waves were shown to be smaller than 300 nm [16]. Furthermore, these waves are believed to play a key role in cellular motility but also in probing of the surrounding matrix, endocytosis and internalization of membrane receptors [19].

In fact, these membrane waves were described for single migrating cells. However, *in vivo*, several fundamental processes require the coordinated motion of cell groups. This collective cell migration plays indeed a key role in developmental processes like gastrulation and organogenesis [20]. Collective movement requires cells to retain cell-cell contacts, exhibit group polarization with defined front-rear asymmetry, and consequently move as one multicellular unit. Depending on the cell type, morphology of the group and the tissue context, distinct mechanisms control the advancing front edge dynamics and guidance. Leading edge migration may either result from adhesion to extracellular matrix and contractile pulling, or from forward pushing. The leading edge consists of either one or few dedicated tip cells (leader cells) or a multicellular leading row that generate adhesion and traction towards the tissue substrate [21]. During development, for example, mesenchymal cells can move in cohorts in a collective manner to their destination. Their behavior is often orchestrated by a collective signal, which might require that all cells have access to guidance information and the ability to interpret this information individually. Alternatively, coordination of mesenchymal migration might be achieved by only selected cells that read a signal and then instruct other cells to follow them by relaying the guidance information to follower cells through chemical or mechanical signaling [22]. In some situations, a mixture of these two signals might operate [23]. We note that to date there are no studies on the role of membrane waves in collective cell migration. In fact, first a more detailed characterization of the membrane waves in individual cell motion is necessary. This can then pave the way for the understanding of their role in collective migration. Furthermore, the transmission of the motion of single cells to the collective motion of many cells has not been extensively studied. The study of the propagation of these waves in single and collective cell motion may reveal their potential role in the mechanism of motility transmission and guidance relevant for the coordinated motility of group of cells.

In this article, we characterize the membrane waves in single and collective cell motion and describe their role in motility transmission among migrating cells for coordinated migration. We perform this by using a superresolution in depth microscopy technique called “optical profilometry” [16], [24]–[26] which provides with our configuration [27], [28] a depth profiling accuracy of around 1,2 nm and lateral resolution of about 200 nm (**figure S1**). Because the optical profilometry technique profiles sample surface by light waves, it does not induce variations of membrane topography. Here, we characterize the membrane waves on single and collective-mesenchymal pre-osteoblast cell migration under the influence of the BMP-2 growth factor that increases cell migration velocity. The choice of this experimental model can contribute to a physiological understanding of cell migration. We finally emphasize the importance of the transmission of these waves among many cells in the description of the dynamic behavior in collective cell motility. The results have significant implications for understanding the mechanistic effects of the *in vivo* microenvironment and also for the therapeutic use of BMPs for the regeneration of skin tissue.

## Results and Discussion

**Figure 1:**
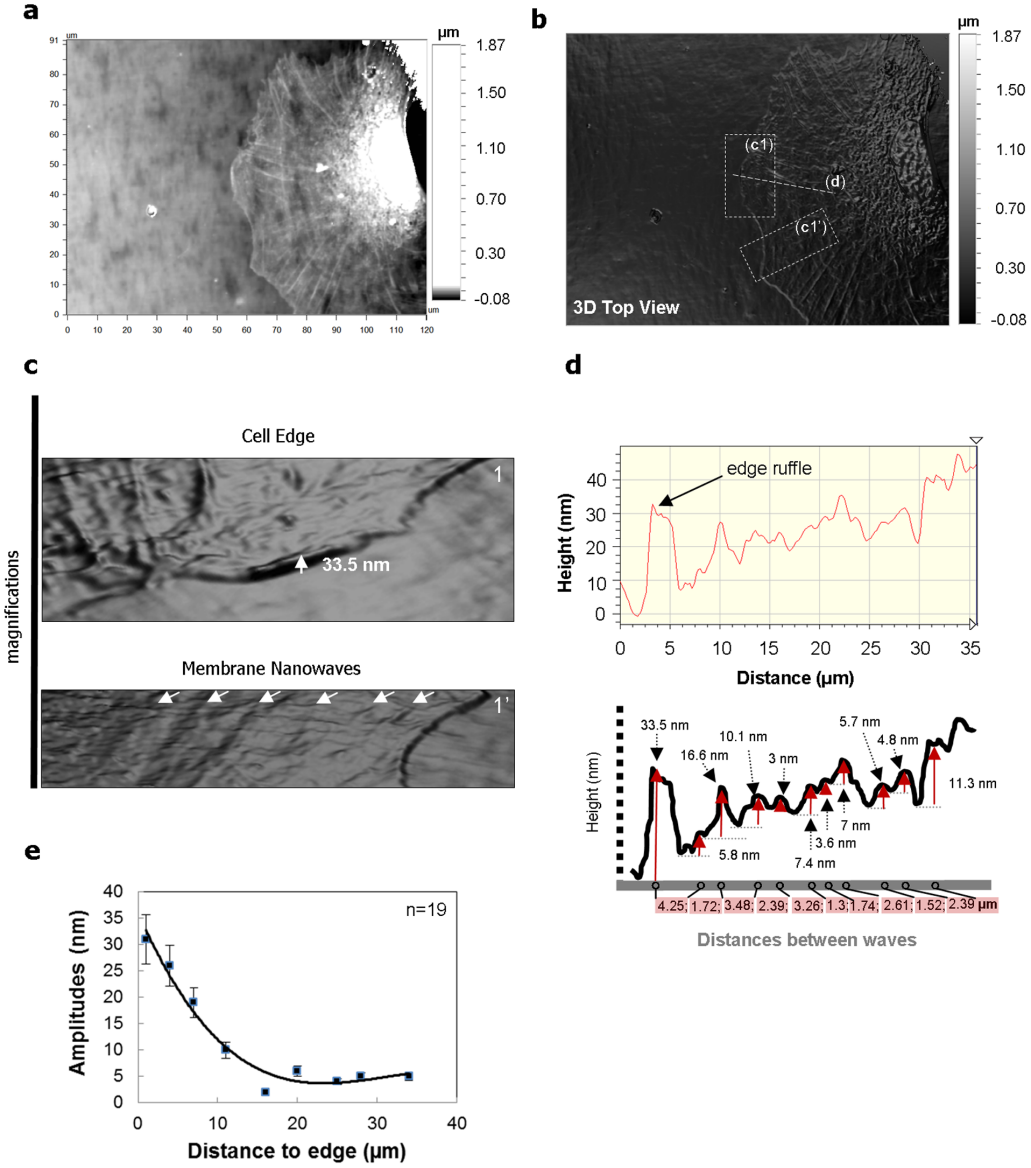
Membrane nanowaves measured by superresolution surface optical profilometry technique. (a) Surface optical profilometry technique micrograph showing a single-cell migration. (b) Surface optical profilometry technique topography 3D reconstruction of a single-cell migration (Top view). (c) Magnifications show cell edge and typical membrane nanowaves nanotopography on pre-osteoblast cells. (d) Membrane-height profile along the migrated cell. In down, details of membrane nanowaves dimensions measurements. (e) Peak-to-valley amplitudes of membranes nanowaves versus the distances to cell edges (n = 19).

Although the membranes can be labeled by lipid-associated dyes and then observed with confocal or two-photon microscopy [29], [30], the height variations in membrane topography are usually smaller than the axial resolution of these optical sectioning techniques. Atomic force microscopy (AFM) has become a regular tool for studies of cell membranes. But owing to the piconeweton force exerted by the tip, AFM measurements usually result from the coupled properties of membranes and cytoskeletons. The interaction force between the membrane and the tip must also be taken into account for correct interpretations of the measurements [31]. In this work, optical profilometry technique was used. In addition to its nanometer resolution, the optical profilometery technique used here does not require external contact with the cell membrane, which is a desirable feature for such nanotopography cell studies. In fact, surface optical profilometry is a non-contact, non-invasive technique suited to the analysis of the evolution of the cell status during biological processes such as migration or differentiation. Herein, we used this technique with phase-shifting interferometry mode for measuring smooth surfaces and steps with nanometer resolution. In this mode, a white-light beam is filtered and passed through an interferometer objective to the test surface. The interferometer beamsplitter reflects half of the incident beam to the reference surface within the interferometer. The beams reflected from the test surface and the reference surface recombine to form interferences fringes. These fringes are alternating light and dark bands when the surface is in focus. **Figure S2** illustrates typical micrographs with interferences fringes for stationary and migrating cells (**figure S2a and b**). The experiments were conducted on mesenchymal pre-osteoblastic MC3T3-E1 cells that have a fibroblastic phenotype [32]. **Figure 1** shows a typical single-cell membrane topography obtained by optical profilometry technique (**figure 1a and b**). Typical three-dimensional membrane waves are highlighted in the magnifications of **figure 1c** and **figure S2c and d**. Concentric membrane waves are clear and the amplitudes gradually decrease during their propagation towards the cell nucleus. The membrane-height profile is depicted in figure 1d. In single migrating cells, we reveal the existence of small and large membrane waves the amplitudes of which are about 3–7 nm to 16–25 nm respectively. Therefore, we will further call these waves membrane nanowaves in this article. The mean wave amplitude is around 32 nm near the cell edge and about 18 nm at a distance of 9 µm away from the edge. This amplitude then decreases to nearly 6 nm with a propagation distance of 31 nm away from the edge (**figure 1e**). At distances higher than 35 nm the nanowaves are not detectable because in the nuclear region the microtubule dynamics disrupts the membrane shape (**figure S3a**).

**Figure 2:**
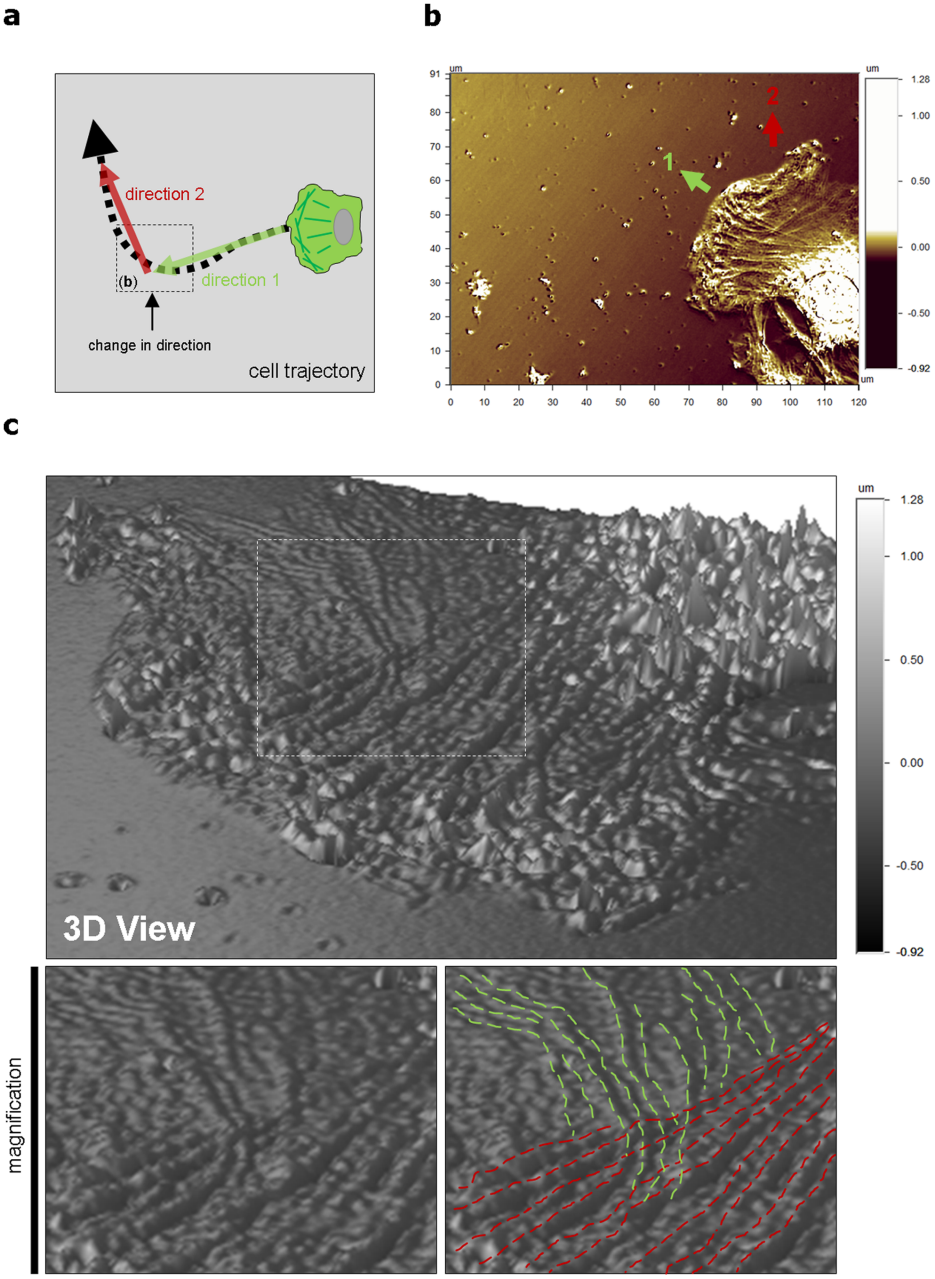
Two membrane nanowaves directions on migrating cells. (a) Schematic drawing of a cell migrating. (b) Surface optical profilometry technique micrograph showing a migrated cell changed its direction. (c) Surface optical profilometry technique 3D view micrograph of this same cell. Magnification shows the two directions in red and green.

Single-pre-osteoblastic cells migrate have a “zig-zag” migration pattern [33] as schematically represented in **figure 2a**. Indeed, these types of cells continuously change the direction of their migration. In this case, we observe interestingly and for the first time the existence of membrane nanowaves of two different orientations in the same cell (**figure 2b, c**). This may participate in the mechanism of the change in direction of the cell motion.

**Figure 3:**
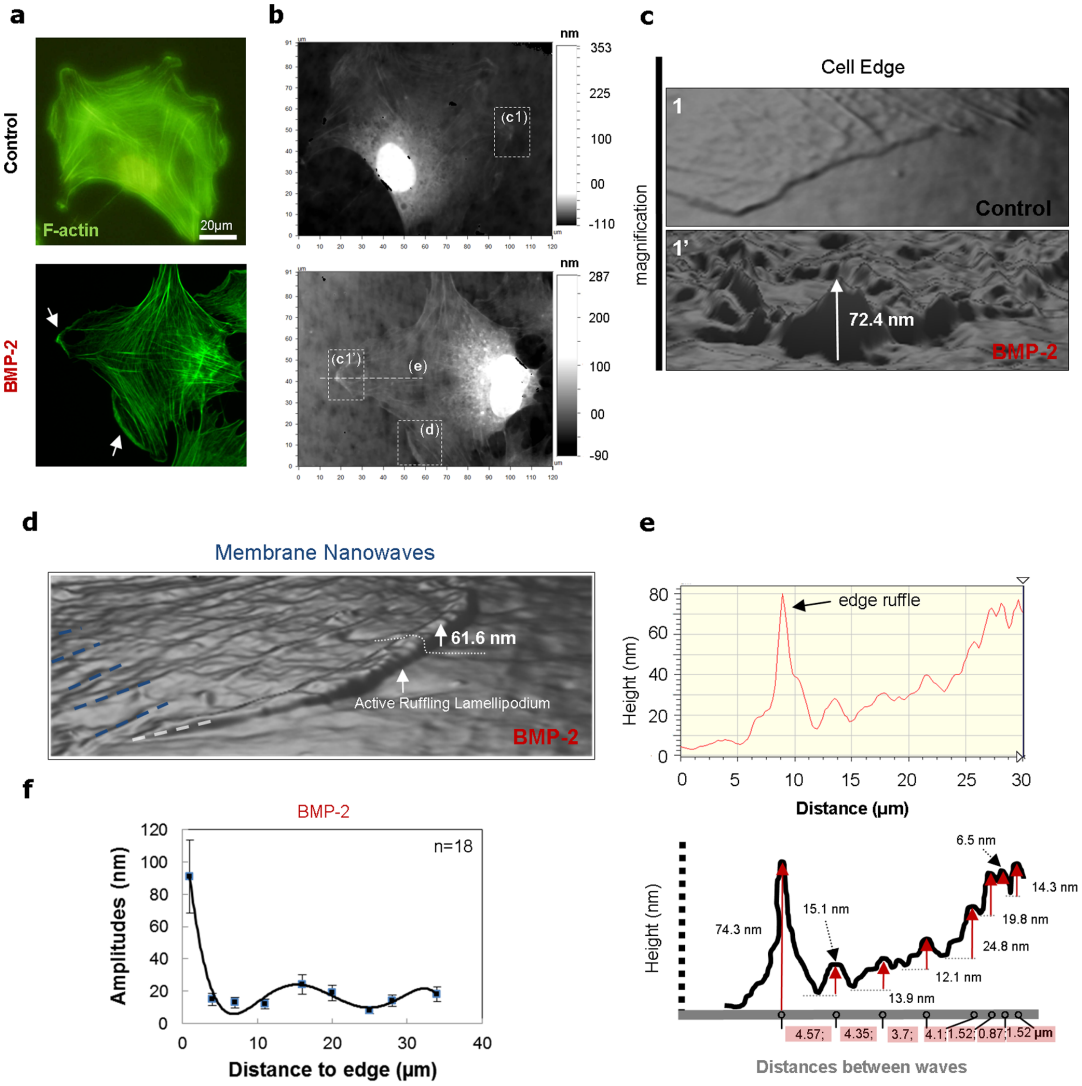
BMP-2 induces cell migration by increasing dimensions of nanowaves. (a) Immunofluorescence staining of migrating pre-osteoblastic cells without and with BMP-2 after culturing for 12 h; the cells were stained for visualization of the actin filaments (green). (b) These same cells are taken with superresolution surface optical profilometry technique. (c) Magnifications show cell edge nanotopography on pre-osteoblast cells treated with BMP-2. (d) Magnifications show the typical membrane nanowaves nanotopography on pre-osteoblast cells treated with BMP-2. (e) Membrane-height profile along the migrated cell treated with BMP-2. In down, details of membrane nanowaves dimensions measurements. (f) Peak-to-valley amplitudes of membranes nanowaves versus the distances to cell edges of migrating cells treated with BMP-2 (n = 18).

Furthermore, as for example in the mammalian body, the mesenchymal pre-osteoblastic cells move into developing and fractured bones along with invading blood vessels participating to their stability [34]. This cell motility is induced by different signal or growth factors in the bone such as the BMPs [35]. As demonstrated in literature [8], BMP-2 increases cell migration. Here, we treated mesenchymal pre-osteoblastic cells with the BMP-2 factor in order to evaluate its influence on the membrane nanowaves characteristics. First, we observed the lamellipodium neo-formation at the cell edges (**figure 3a**, see arrows). The wave’s amplitude at the cell edges increases to about 92 nm compared to cells not treated with BMP-2 (**figure 3b, c**). We also show the active ruffling lamellipodium followed by the membrane nanowaves (**figure 3d**). We show the typical membrane-height profile of migrating pre-osteoblasts treated with BMP-2 in **figure 3e**. The average amplitudes are about 15 nm at a distance of 9 µm from the cell edge. Contrary to the non-treated cells, the amplitudes of the waves are then stabilized to around 18 nm with a propagation distance of 31 µm away from the cell edge (**figure 3f**). No change in the direction of migration was observed in the case of pre-osteoblasts treated with BMP-2. Furthermore only nanowaves of a single orientation were observed in the same cell. In summary, BMP-2 increases the dimensions of the membrane nanowaves and also inhibits the formation of nanowaves of different orientations in the same cell and the change in the migration direction.

**Figure 4:**
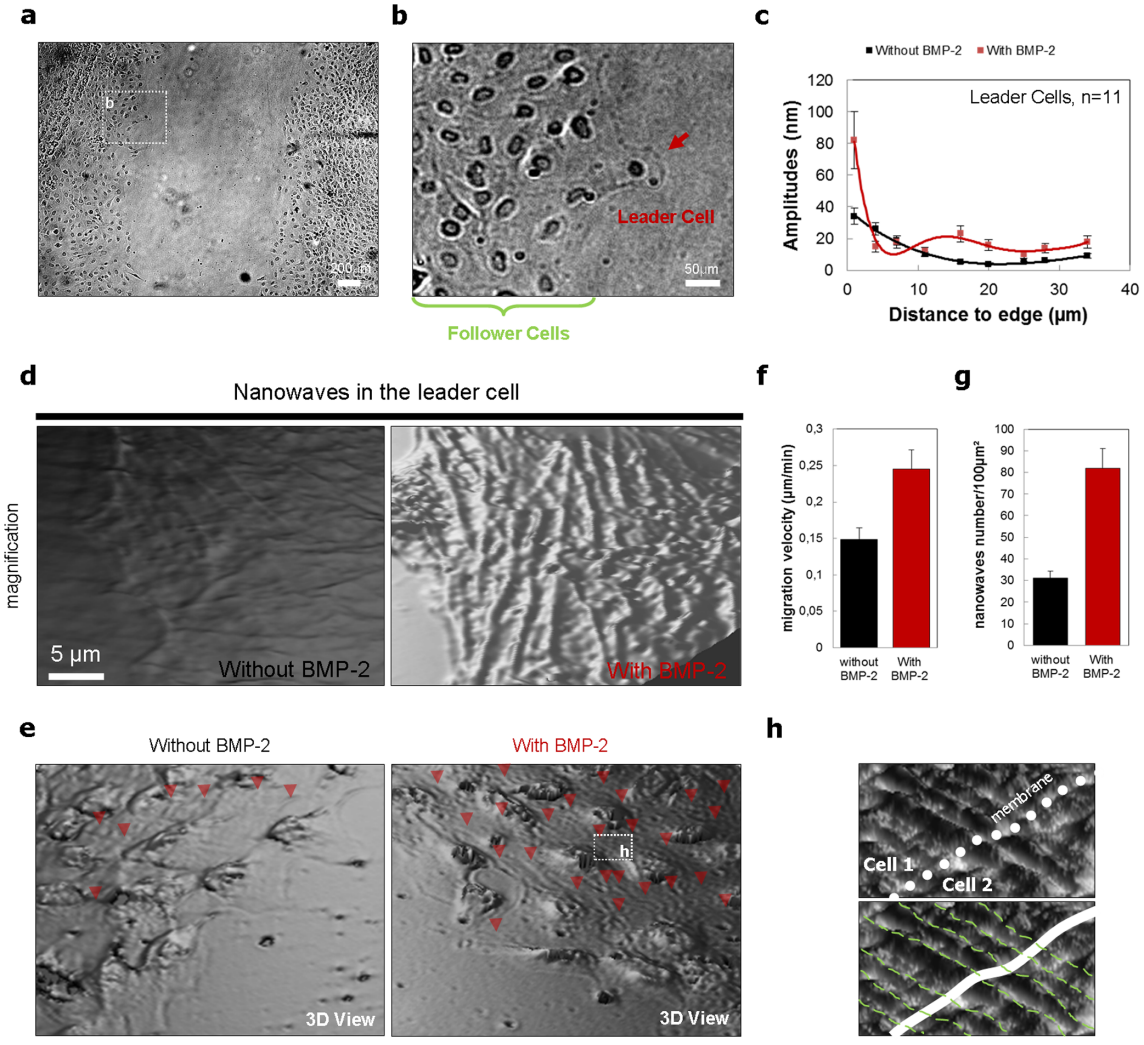
Membrane nanowaves generation and propagation in collective-cell migration. (a) Phase-contrast Micrograph showing the progression of migrating cells after scratching. (b) Phase-contrast Micrograph of leader cell 12 h after scratching. (c) Peak-to-valley amplitudes of membranes nanowaves versus the distances to cell edges of migrating leader cells treated or not with BMP-2 (n = 11). (d) Magnification shows cell edge and membrane nanowaves of leader cells treated or not with BMP-2. There is a very active, ruffling lamellipodium. (e) Surface optical profilometry technique 3D view micrograph of collective-cell migration without and with BMP-2 treatment. (f) Mean pre-osteoblast cell velocity measured with and without BMP-2 treatment. (g) Graph showing membrane nanowaves number per 100 µm^2^ with and without BMP-2 treatment. (h) Surface optical profilometry technique 3D view micrograph magnification of membrane waves shared by two migrating cells in the case of BMP-2 treatment.

The single-cell motion comprehension with membrane wave’s changes encourages us to study collective-cell motion and guidance with this same parameter. In fact, we applied a usual scratch test [36] on a monolayer of mesenchymal pre-osteoblast cells in culture (**figure 4a**). The cells then migrate collectively and the communication between cells is maintained. Typically, after 1 hour, we observed the appearance of “leader cells” [21], [37] which were very distinct from the other cells in the migration front (**figure 4b**) as they were much larger and spread out. These cells developed a clear active ruffling lamellipodium with a loss of their initial morphology. However, they maintained cell-cell contacts with their “followers”. The leader cells are characterized by membrane waves with amplitude of around 34 nm near the cell edge, of about 20 nm at a distance of 9 µm, which then decreases to nearly 7 nm with a propagation distance of 31 µm (**figure 4c, d**). Some of these membrane nanowaves were transmitted from the leader to the follower cells (**figure 4e**). The collective-cell migration for cells treated with BMP-2 shows increased velocity (**figure 4f**). In this case the leader cells are characterized by membrane waves with amplitude of around 83 nm near the cell edge and around 24 nm at a distance of 9 µm. Contrary to the control, the amplitude is stabilized to nearly 19 nm with a propagation distance of 31 µm (**figure 4c, d**). Surprisingly, the membrane nanowaves are transmitted from leader to several rows follower cells (**figure 4e**). The quantification of the number of membrane nanowaves for cells treated or no with BMP-2 further supports this suggestion (**figure 4g**). The transmission of membrane nanowaves between cells may also be part of the mechanism of synchronization of collective-cell motion. In fact, the vast majority of the observed membrane nanowaves is shared between the adjacent cells (**figure 4h** and **figure S4**). In summary for collectively migrating cells the membrane nanowaves are shared by neighboring migrating cells. As previously demonstrated, collective-cell migration velocity is controlled by cell-cell interactions via adherens junctions [38]. Here we also demonstrate that collective-cell motion is also controlled by membrane nanotopography communications. This work suggests that maintaining collectivity during cell migration may be governed by “intimate contact” via the membrane nanowaves sharing.

Recent advances have revealed that intercellular direct transmission of physical forces can coordinate collective cell motion [39]. Otherwise, mechanical waves that propagate slowly to span the monolayer traverse intercellular junctions in a cooperative manner, were also described to explain collective cell motion [39], [40]. Very interesting observations demonstrate that the combination of growing dynamic heterogeneities and slowing migration speed with increasing cell density is strikingly reminiscent of the nature of the relaxations observed in supercooled fluids approaching the glass transition. This suggests the possibility of an analogy between this cell motion within a confluent layer and the crowding within a particulate system approaching a glass transition with increasing density [41]. Our study here provides an explanation of collective cell guidance by propagation of membrane nanowaves from leader to follower cells. All these *in vitro* observations could explain different physiological functions including morphogenesis and tissue regeneration.

As demonstrated previously theoretically and experimentally, contractile force of Myosin II and the protrusion force from actin polymerization are required for the generation of membrane waves [15], [16], [42]. Sheetz and co-workers’ study has revealed that myosin pulls the rear of the lamellipodial actin network (which appears to sit above the lamellum), causing upward bending and edge retraction. The network then separates from the edge and condenses. Protrusion resumes as lamellipodial actin regenerates from the front and extends rearward until it reaches newly assembled myosin, initiating the next cycle. Thus, actin polymerization periodically builds the lamellipodium, connecting myosin motors with the initiation of adhesion sites, suggesting that the actin oscillator and retrograde waves are mechanical in nature [15]. More recently, Gov and co-workers’ proposed a theoretical model which attributes the formation of membrane waves to the interplay between complexes that contain activators of actin polymerization and membrane-bound curved proteins of both types of curvature (concave and convex) [43]. Experimentally, they show that waves have been associated with membrane bound activators of actin polymerization of concave shape. The convex membrane proteins are insensitive to inhibition of myosin II contractility [43].

It is becoming clear that membrane waves result from activation and inhibition feedbacks in actin dynamics acting on different scales, but the exact molecular nature of these feedbacks and the respective roles of biomechanics and biochemistry are still unclear. Moreover, the relationship between these membrane nanowaves and the mechanical waves recently observed by the group of Trepat X. [40] is still unknown. Our work demonstrates the direct cell-cell cooperation by sharing membrane waves. This new role attributed to the membrane waves raises the question of whether a cooperation exists between the adherent junctions, the cell cytoskeleton and the membrane waves and their interregulation. Future experiments and physical models can answer these attractive questions.

## Conclusions

In summary, we used the wide-field optical profiling technique with nanometer resolution to characterize the three-dimensional membrane waves and their role in single and collective cell migration. The membrane waves investigated on mesenchymal pre-osteoblastic cells have nanodimension size. The treatment with the BMP-2 growth factor changes effectively the characteristics of these membrane nanowaves like their amplitude at the cell edge and their distance propagation. This can explain the guidance of collective-cell migration through leader cells. We suggest that collective cell migration velocity is controlled by cell-cell interactions that are governed by membrane topography communications. More generally, this work also suggests that cellular functions in particularly migration could be governed by external mechanical properties *via* membrane nanowaves. Furthermore, in the future, the surface optical profilometry technique used here can provide more information regarding the mechanisms of other cellular functions such as differentiation by monitoring changes in the cell membrane nanotopography. The results have significant implications for understanding the mechanistic effects of the *in vivo* microenvironment and also for the therapeutic use of BMPs for the regeneration of skin tissue.

## Experimental Procedures

### Cell Culture

Mouse mesenchymal pre-osteoblastic cells [32] (MC3T3-E1, from ATCC) were cultured in Alpha-MEM medium supplemented with 10% fetal calf serum (FCS) and 1% penicillin/streptomycin. All cells were used at a low passage number (passage 4), and subconfluent cultures were used; the cells were plated at 10,000 cells/cm^2^ for experiments. For the soluble BMP-2 protein induction mode, the pre-osteoblast cells were cultured on plastic substrates and were exposed to 300 ng/mL of recombinant human BMP-2 (Peprotech)-containing media for 1 to 12 hours. For superresolution surface optical profilometry technique observations, the samples were dehydrated in increasing concentrations of ethanol (30, 70, 80, 90, 95 and 100%) and critical-point dried. Finally, the samples were metallized for 10 sec with gold or titanium were examined. This protocol doesn’t affect the cell shape and dimension. We have previously induced both shrinkage and swelling of different type of cells (mesenchymal stem cells, osteoblast and endothelial cells) and have thus demonstrated that superresolution surface optical profilometry technique does not induce any artifacts on the performed measurements. Three selected examples are listed and briefly presented here to show our expertise of this technique. In the first example, we have developed a differentiation model and have characterized differentiated cells by using this superresolution wide-field optical profilometry microscopy technique. We have induced cell swelling and have demonstrated that the cell fixation method (which also involves gold or titanium deposition) does not alter the surface topography to any significant degree thus ruling out any artifacts [32], [44]. In a second example, we have developed another nanotopographical model for stem cell differentiation and have analyzed cell adhesion and collective cell organization by using the same superresolution technique. We have proposed a functional model for stem cell differentiation through the adhesion process and the cytoskeleton organization [27]. Finally, we have also used this technique to characterize lamellipodial and filopodial cell migration [28]. In all of these examples, we have performed induction of different types of cells towards different cell statuses with both shrinkage and swelling. Our protocol (fixation, dehydration and gold coating) does not modify the cell morphology or cell shape. The superresolution technique used here is very sensitive but also suitable to study the cell membrane waves. During the drawing of the profile of waves, all the disturbances (more specifically, the actin stress fibers) have been eliminated. The actin stress fibers were removed by superimposing the surface optical profilometry technique profile and the F-actin immunostaining profile. Analyzed cell-surface topography was only the physical deformation of representative membrane waves as they have been previously defined in literature [16], [42].

### Immunostaining

As previously described [27], [45], [46], after 1 to 12 hours in culture, the cells on the plastic surfaces were fixed for 20 min in 4% paraformaldehyde/PBS at 4°C. After fixation, the cells were permeabilized in PBS 1X containing 1% Triton X-100 for 15 min. The cytoskeletal filamentous actin (F-actin) was visualized by treating the cells with 5 U/mL Alexa Fluor 488 phalloidin (Sigma, France) for 1 h at 37°C. The images for this experiment were produced using a Leica SP5 microscope and MetaMorph software.

### The Surface Optical Profilometry Technique

As described previously [27], [28], [32], the surface profiler system is non-contact optical profilers that use two technologies to measure a wide range of surface heights. The phase-shifting interferometry (PSI) mode allows for the measurement of smooth surfaces and small steps, whereas the vertical scanning interferometry (VSI) mode allows for the measurement of rough surfaces and steps up to several micrometers high. In this work, we used only PSI mode to characterize membrane waves. The light for both modes originates from a white-light source; however, it is filtered during PSI measurements to produce red light at a nominal wavelength of 632 nm. In this PSI mode, the objective does not move through focus. Instead, we focus on the sample so the region of interest is at precise focus, then we make the measurement. During the measurements, the piezoelectric transducer (PZT) causes a slight shift between the reference and sample beams. The measurement is very quick. For the calculation of the cell membrane waves, the reference is different for each wave. In fact, the reference is the point that the wave starts (**figure S3b and c**). Moreover, a fluorescence microscopy was adapted for the optical profilometer configuration. A superresolution optical profilometry microscopy technique provides with our configuration [27], [47] a depth profiling accuracy of around 1,2 nm and lateral resolution of about 200 nm. We have determined the vertical resolution of our system by taking the difference of two measurements from the same location on the sample. The resolution data is essentially the noise limit of our system. It should be a near-flat profile with R_q_ value approaching the non-averaged values. To get the best nanometer resolution from optical profilometry microscopy technique, consistent and correct measurements have been used. **Figure S1** shows the example amplitude parameters (R_a_ and R_q_) obtained in our control surfaces. R_a_ represents the *roughness average*, the arithmetic mean of the absolute values of the surface departures from the mean plane. The digital approximation for three-dimensional R_a_ is:
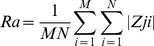
Where M and N are the number of data points in the X and Y direction, respectively, of the array, and Z is the surface height relative to the reference mean plane.

R_q_ represents the *root mean square roughness*, obtained by squaring each height value in the dataset, then taking the square root of the mean. The digital approximation for the three-dimensional R_q_ is given by:
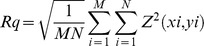
R_q_ has statistical significance because it represents the standard deviation of the surface heights, and it is used in the more complex computations of *skewness* (briefly, R_sk_ that measures the asymmetry of the surface about the mean plane) and *kurtosis* (briefly, R_ku_ that measures the peakedness of the surface about the mean plane).

In our work, we characterize the three-dimensional membrane waves for migrating single and collective cells and describe their propagation using a superresolution wide-field optical profiling technique as described by Reed *et al* [48]. The goal of this study is not to prove the existence of membrane waves but to apply a new protocol coupled to this method in order to obtain cell-surface high resolution in the context of single and collective cell migration. Based on our observations reported in this work, we propose for the first time a role for these membrane waves. The results and conclusions of this article can thus contribute to the mechanistic study of cell migration.

### Statistical Analysis

In terms of cell velocity, the data were expressed as the mean ± standard error, and were analyzed using the paired Student’s t-test method. Significant differences were designated for *P* values of at least <0.01.

## Supporting Information

Figure S1 (a) shows a surface optical profilometry technique micrographs showing a control surface. Ra is about 1.38 and Rq about 1.83 for this image. (b) shows a surface optical profilometry technique 3D reconstruction of the control surface.
(TIF)

Figure S2 (a) and (b) shows a surface optical profilometry technique micrographs with high-contrast fringe mode showing a single-cell on two different stationary and migration status respectively. (c) Surface optical profilometry technique topography 3D reconstruction of a single-cell migration. (d) Magnification showing typical membrane nanowaves nanotopography on pre-osteoblast cells (see red lines and white arrows).
(TIF)

Figure S3 (a) Surface optical profilometry technique topography 3D reconstruction of a single-cell migration. (b) Surface optical profilometry technique micrograph showing a single-cell migration. (c) Membrane-height profile of the red line on the migrated cell.
(TIF)

Figure S4 Surface optical profilometry technique 2D (a) and 3D view micrographs (b) of collective cell migration with BMP-2 treatment. We show membrane nanowaves directions (small white arrows: nanowaves, big white arrows: direction of nanowaves).
(TIF)

## References

[1] Martin P, Parkhurst SM (2004) Parallels between tissue repair and embryo morphogenesis. Development 131: 3021–3034. 15197160 10.1242/dev.01253

[2] Lecaudey V, Gilmour D (2006) Organizing moving groups during morphogenesis. Curr Opin Cell Biol 18: 102–107. 16352429 10.1016/j.ceb.2005.12.001

[3] Friedl P, Hegerfeldt Y, Tusch M (2004) Collective cell migration in morphogenesis and cancer. Int J Dev Biol 48: 441–449. 15349818 10.1387/ijdb.041821pf

[4] Stramer B, Wood W, Galko MJ, Redd MJ, Jacinto A, et al (2005) Live imaging of wound inflammation in Drosophila embryos reveals key roles for small GTPases during in vivo cell migration. J Cell Biol 168: 567–573. 15699212 10.1083/jcb.200405120 PMC2171743

[5] Ridley AJ (2011) Life at the leading edge. Cell 145: 1012–1022. 21703446 10.1016/j.cell.2011.06.010

[6] Bugyi B, Carlier MF (2010) Control of actin filament treadmilling in cell motility. Annu Rev Biophys 39: 449–470. 20192778 10.1146/annurev-biophys-051309-103849

[7] Bergert M, Chandradoss SD, Desai RA, Paluch E (2012) Cell mechanics control rapid transitions between blebs and lamellipodia during migration. Proc Natl Acad Sci U S A 109: 14434–14439. 22786929 10.1073/pnas.1207968109 PMC3437886

[8] Gamell C, Osses N, Bartrons R, Ruckle T, Camps M, et al (2008) BMP2 induction of actin cytoskeleton reorganization and cell migration requires PI3-kinase and Cdc42 activity. J Cell Sci 121: 3960–3970. 19001503 10.1242/jcs.031286

[9] Inai K, Norris RA, Hoffman S, Markwald RR, Sugi Y (2008) BMP-2 induces cell migration and periostin expression during atrioventricular valvulogenesis. Dev Biol 315: 383–396. 18261719 10.1016/j.ydbio.2007.12.028 PMC3644399

[10] Sieber C, Kopf J, Hiepen C, Knaus P (2009) Recent advances in BMP receptor signaling. Cytokine Growth Factor Rev 20: 343–355. 19897402 10.1016/j.cytogfr.2009.10.007

[11] Das RK, Zouani OF (2014) A review of the effects of the cell environment physicochemical nanoarchitecture on stem cell commitment. Biomaterials. 10.1016/j.biomaterials.2014.03.044 24720880

[12] Kaibuchi K, Kuroda S, Amano M (1999) Regulation of the cytoskeleton and cell adhesion by the Rho family GTPases in mammalian cells. Annu Rev Biochem 68: 459–486. 10872457 10.1146/annurev.biochem.68.1.459

[13] Dobereiner HG, Dubin-Thaler BJ, Hofman JM, Xenias HS, Sims TN, et al (2006) Lateral membrane waves constitute a universal dynamic pattern of motile cells. Phys Rev Lett 97: 038102. 16907546 10.1103/PhysRevLett.97.038102

[14] Giannone G, Dubin-Thaler BJ, Dobereiner HG, Kieffer N, Bresnick AR, et al (2004) Periodic lamellipodial contractions correlate with rearward actin waves. Cell 116: 431–443. 15016377 10.1016/s0092-8674(04)00058-3

[15] Giannone G, Dubin-Thaler BJ, Rossier O, Cai Y, Chaga O, et al (2007) Lamellipodial actin mechanically links myosin activity with adhesion-site formation. Cell 128: 561–575. 17289574 10.1016/j.cell.2006.12.039 PMC5219974

[16] Chen CH, Tsai FC, Wang CC, Lee CH (2009) Three-dimensional characterization of active membrane waves on living cells. Phys Rev Lett 103: 238101. 20366177 10.1103/PhysRevLett.103.238101

[17] Machacek M, Danuser G (2006) Morphodynamic profiling of protrusion phenotypes. Biophys J 90: 1439–1452. 16326902 10.1529/biophysj.105.070383 PMC1367294

[18] Coelho Neto J, Agero U, Oliveira DC, Gazzinelli RT, Mesquita ON (2005) Real-time measurements of membrane surface dynamics on macrophages and the phagocytosis of Leishmania parasites. Exp Cell Res 303: 207–217. 15652336 10.1016/j.yexcr.2004.09.002

[19] Buccione R, Orth JD, McNiven MA (2004) Foot and mouth: podosomes, invadopodia and circular dorsal ruffles. Nat Rev Mol Cell Biol 5: 647–657. 15366708 10.1038/nrm1436

[20] Tam PP, Behringer RR (1997) Mouse gastrulation: the formation of a mammalian body plan. Mech Dev 68: 3–25. 9431800 10.1016/s0925-4773(97)00123-8

[21] Khalil AA, Friedl P (2010) Determinants of leader cells in collective cell migration. Integr Biol (Camb) 2: 568–574. 20886167 10.1039/c0ib00052c

[22] Zouani OF, Lei Y, Durrieu MC (2013) Pericytes, stem-cell-like cells, but not mesenchymal stem cells are recruited to support microvascular tube stabilization. Small 9: 3070–3075. 23625793 10.1002/smll.201300124

[23] Weijer CJ (2009) Collective cell migration in development. J Cell Sci 122: 3215–3223. 19726631 10.1242/jcs.036517

[24] Lee CH, Mong HY, Lin WC (2002) Noninterferometric wide-field optical profilometry with nanometer depth resolution. Opt Lett 27: 1773–1775. 18033359 10.1364/ol.27.001773

[25] Wang CC, Lin JY, Chen HC, Lee CH (2006) Dynamics of cell membranes and the underlying cytoskeletons observed by noninterferometric widefield optical profilometry and fluorescence microscopy. Opt Lett 31: 2873–2875. 16969407 10.1364/ol.31.002873

[26] Hsu TH, Liao WY, Yang PC, Wang CC, Xiao JL, et al (2007) Dynamics of cancer cell filopodia characterized by super-resolution bright-field optical microscopy. Opt Express 15: 76–82. 19532223 10.1364/oe.15.000076

[27] Zouani OF, Chanseau C, Brouillaud B, Bareille R, Deliane F, et al (2012) Altered nanofeature size dictates stem cell differentiation. J Cell Sci 125: 1217–1224. 22302989 10.1242/jcs.093229

[28] Lei Y, Zouani OF, Rami L, Chanseau C, Durrieu MC (2013) Modulation of lumen formation by microgeometrical bioactive cues and migration mode of actin machinery. Small 9: 1086–1095. 23161822 10.1002/smll.201202410

[29] Schutz GJ, Kada G, Pastushenko VP, Schindler H (2000) Properties of lipid microdomains in a muscle cell membrane visualized by single molecule microscopy. EMBO J 19: 892–901. 10698931 10.1093/emboj/19.5.892 PMC305629

[30] Gaus K, Gratton E, Kable EP, Jones AS, Gelissen I, et al (2003) Visualizing lipid structure and raft domains in living cells with two-photon microscopy. Proc Natl Acad Sci U S A 100: 15554–15559. 14673117 10.1073/pnas.2534386100 PMC307606

[31] Sen S, Subramanian S, Discher DE (2005) Indentation and adhesive probing of a cell membrane with AFM: theoretical model and experiments. Biophys J 89: 3203–3213. 16113121 10.1529/biophysj.105.063826 PMC1366816

[32] Zouani OF, Chollet C, Guillotin B, Durrieu MC (2010) Differentiation of pre-osteoblast cells on poly(ethylene terephthalate) grafted with RGD and/or BMPs mimetic peptides. Biomaterials 31: 8245–8253. 20667411 10.1016/j.biomaterials.2010.07.042

[33] Driscoll MK, McCann C, Kopace R, Homan T, Fourkas JT, et al (2012) Cell shape dynamics: from waves to migration. PLoS Comput Biol 8: e1002392. 22438794 10.1371/journal.pcbi.1002392 PMC3305346

[34] Maes C, Kobayashi T, Selig MK, Torrekens S, Roth SI, et al (2010) Osteoblast precursors, but not mature osteoblasts, move into developing and fractured bones along with invading blood vessels. Dev Cell 19: 329–344. 20708594 10.1016/j.devcel.2010.07.010 PMC3540406

[35] Wagner DO, Sieber C, Bhushan R, Borgermann JH, Graf D, et al (2010) BMPs: from bone to body morphogenetic proteins. Sci Signal 3: mr1. 20124549 10.1126/scisignal.3107mr1

[36] Liang CC, Park AY, Guan JL (2007) In vitro scratch assay: a convenient and inexpensive method for analysis of cell migration in vitro. Nat Protoc 2: 329–333. 17406593 10.1038/nprot.2007.30

[37] Poujade M, Grasland-Mongrain E, Hertzog A, Jouanneau J, Chavrier P, et al (2007) Collective migration of an epithelial monolayer in response to a model wound. Proc Natl Acad Sci U S A 104: 15988–15993. 17905871 10.1073/pnas.0705062104 PMC2042149

[38] Tambe DT, Hardin CC, Angelini TE, Rajendran K, Park CY, et al (2011) Collective cell guidance by cooperative intercellular forces. Nat Mater 10: 469–475. 21602808 10.1038/nmat3025 PMC3135682

[39] Trepat X, Wasserman MR, Angelini TE, Millet E, Weitz DA, et al (2009) Physical forces during collective cell migration. Nat Phys 5: 426–430.

[40] Serra-Picamal X, Conte V, Vincent R, Anon E, Tambe DT, et al (2012) Mechanical waves during tissue expansion. Nat Phys 8: 628–634.

[41] Angelini TE, Hannezo E, Trepat X, Marquez M, Fredberg JJ, et al (2011) Glass-like dynamics of collective cell migration. Proc Natl Acad Sci U S A 108: 4714–4719. 21321233 10.1073/pnas.1010059108 PMC3064326

[42] Shlomovitz R, Gov NS (2007) Membrane waves driven by actin and Myosin. Phys Rev Lett 98: 168103. 17501468 10.1103/PhysRevLett.98.168103

[43] Peleg B, Disanza A, Scita G, Gov N (2011) Propagating cell-membrane waves driven by curved activators of actin polymerization. PLoS One 6: e18635. 21533032 10.1371/journal.pone.0018635 PMC3080874

[44] Zouani OF, Rami L, Lei Y, Durrieu MC (2013) Insights into the osteoblast precursor differentiation towards mature osteoblasts induced by continuous BMP-2 signaling. Biol Open 2: 872–881. 24143273 10.1242/bio.20134986 PMC3773333

[45] Lei Y, Zouani OF, Remy M, Ayela C, Durrieu MC (2012) Geometrical microfeature cues for directing tubulogenesis of endothelial cells. PLoS One 7: e41163. 22829923 10.1371/journal.pone.0041163 PMC3400641

[46] Zouani OF, Kalisky J, Ibarboure E, Durrieu MC (2013) Effect of BMP-2 from matrices of different stiffnesses for the modulation of stem cell fate. Biomaterials 34: 2157–2166. 23290467 10.1016/j.biomaterials.2012.12.007

[47] Lei Y, Zouani OF, Rami L, Chanseau C, Durrieu MC (2012) Modulation of Lumen Formation by Microgeometrical Bioactive Cues and Migration Mode of Actin Machinery. Small. 10.1002/smll.201202410 23161822

[48] Reed J, Troke JJ, Schmit J, Han S, Teitell MA, et al (2008) Live cell interferometry reveals cellular dynamism during force propagation. ACS Nano 2: 841–846. 19206480 10.1021/nn700303f PMC2733939

