# Supplementary figures and images for: Membrane Nanowaves in Single and Collective Cell Migration
Source: PLoS One. 2014 May 20;9(5):e97855. doi: 10.1371/journal.pone.0097855 (PMC4028249; doi:10.1371/journal.pone.0097855)

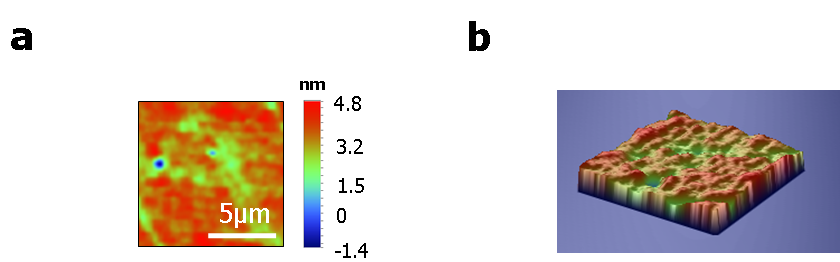

Supplement: Figure S1 — (a) shows a surface optical profilometry technique micrographs showing a control surface. Ra is about 1.38 and Rq about 1.83 for this image. (b) shows a surface optical profilometry technique 3D reconstruction of the control surface. (TIF) [file pone.0097855.s001.tif]

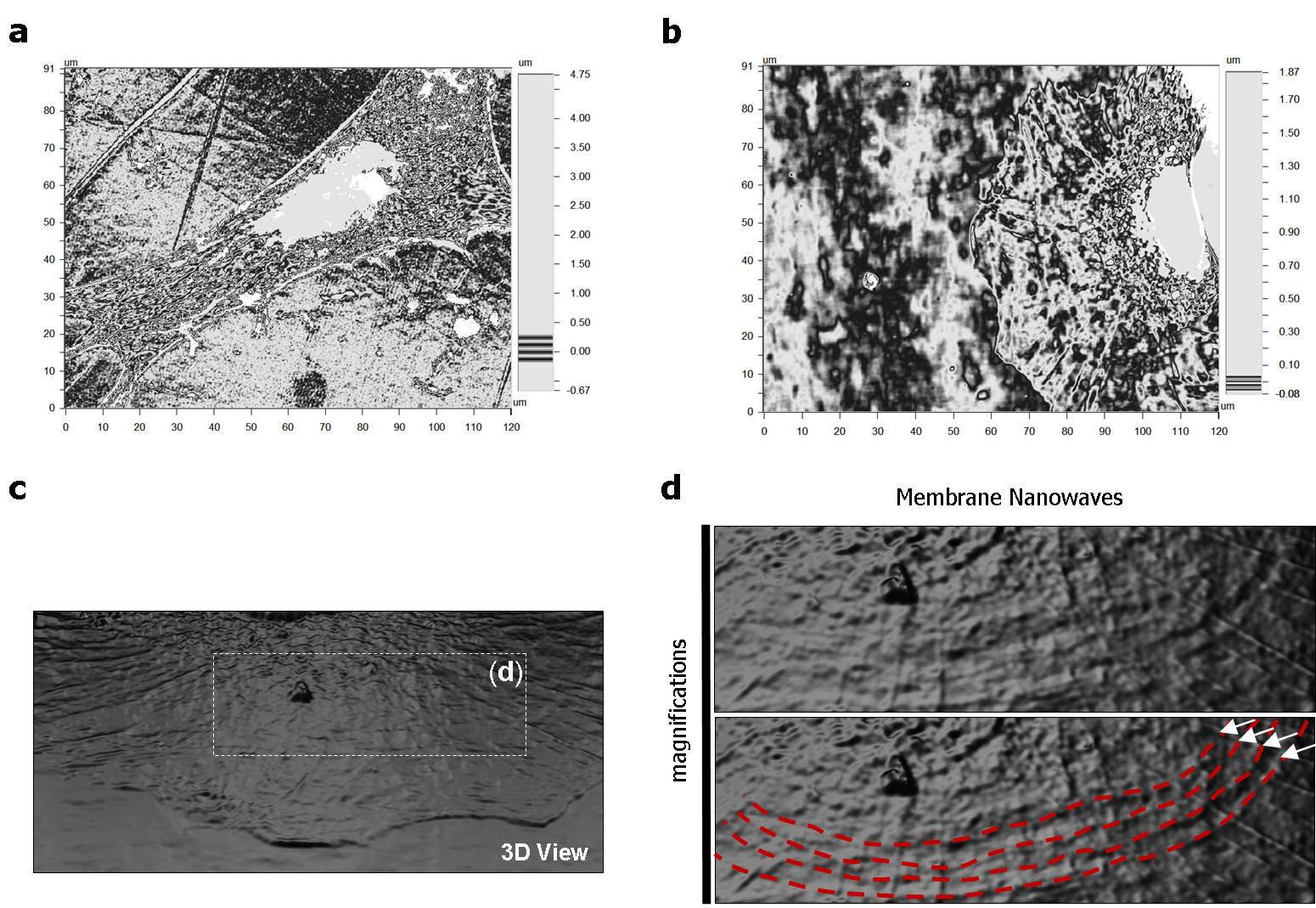

Supplement: Figure S2 — (a) and (b) shows a surface optical profilometry technique micrographs with high-contrast fringe mode showing a single-cell on two different stationary and migration status respectively. (c) Surface optical profilometry technique topography 3D reconstruction of a single-cell migration. (d) Magnification showing typical membrane nanowaves nanotopography on pre-osteoblast cells (see red lines and white arrows). (TIF) [file pone.0097855.s002.tif]

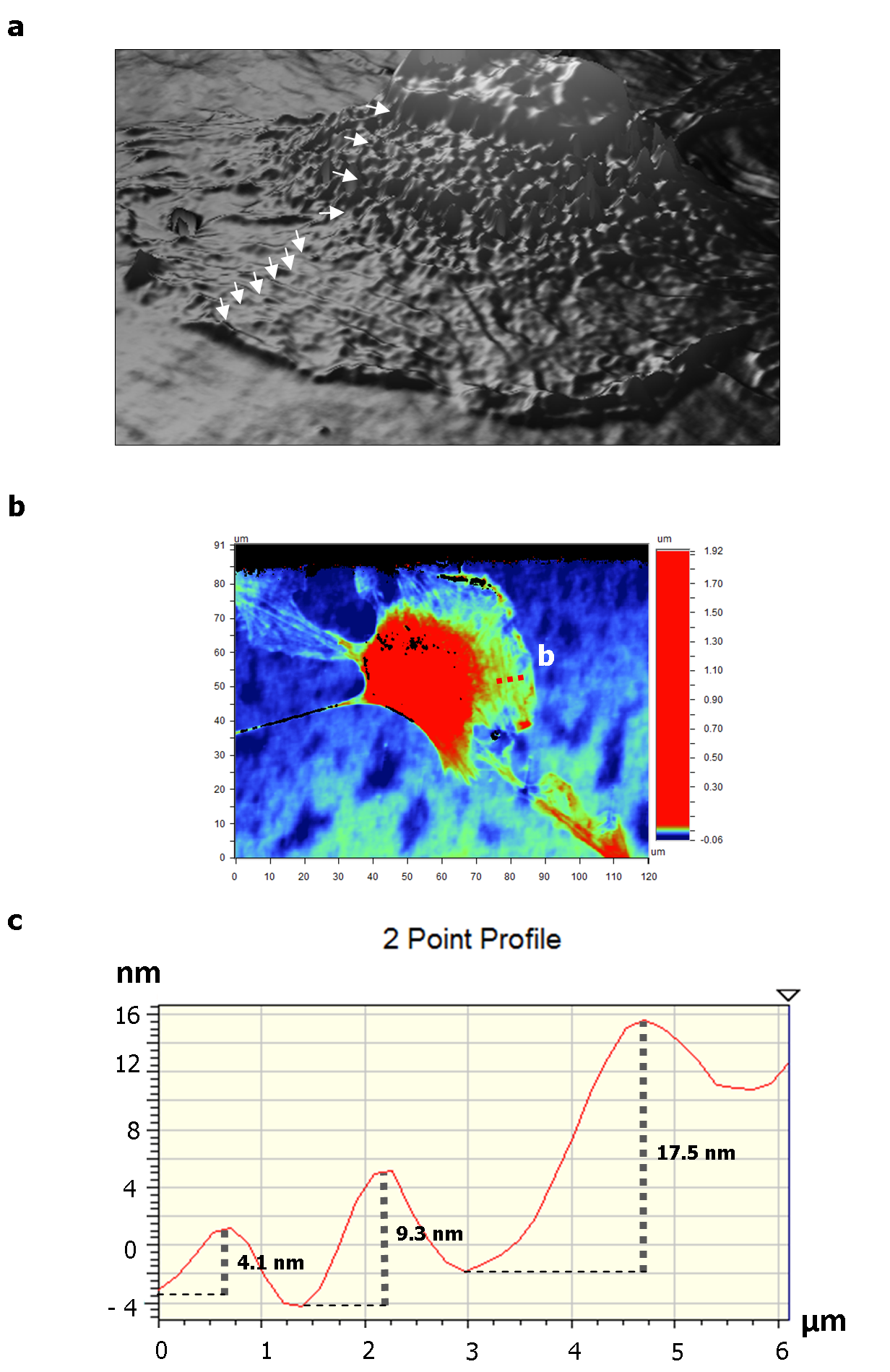

Supplement: Figure S3 — (a) Surface optical profilometry technique topography 3D reconstruction of a single-cell migration. (b) Surface optical profilometry technique micrograph showing a single-cell migration. (c) Membrane-height profile of the red line on the migrated cell. (TIF) [file pone.0097855.s003.tif]

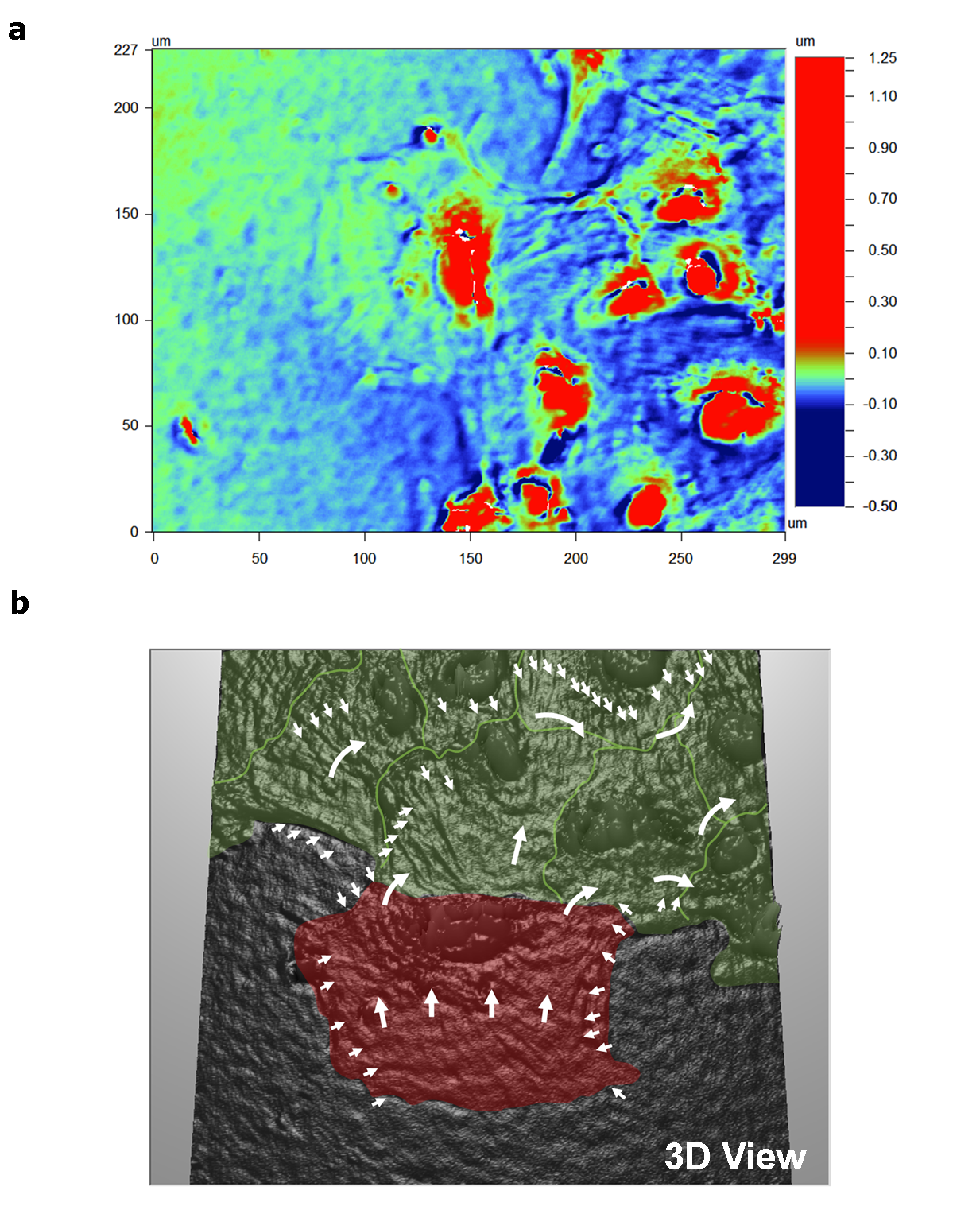

Supplement: Figure S4 — Surface optical profilometry technique 2D (a) and 3D view micrographs (b) of collective cell migration with BMP-2 treatment. We show membrane nanowaves directions (small white arrows: nanowaves, big white arrows: direction of nanowaves). (TIF) [file pone.0097855.s004.tif]
